# Early Open Kinetic Chain Hamstring Exercise After ACL Reconstruction: A Retrospective Safety and Efficacy Study

**DOI:** 10.3390/jcm14196871

**Published:** 2025-09-28

**Authors:** Roberto Ricupito, Rosalba Castellucci, Filippo Maselli, Marco Bravi, Fabio Santacaterina, Riccardo Guarise, Florian Forelli

**Affiliations:** 1 Restart Physiotherapy, Via Felice Grossi Gondi 35, 00162 Rome, Italy; 2 Castellucci Private Clinic, Via Maiella, 75023 Montalbano Jonico, Italy; castellucci.ross@gmail.com; 3 Sovrintendenza Sanitaria Regionale Puglia, INAIL, 70126 Bari, Italy; masellifilippo76@gmail.com; 4 Department of Human Neurosciences, Sapienza University of Rome, 00185 Rome, Italy; 5 Fondazione Policlinico Universitario Campus Bio-Medico, Via Alvaro del Portillo 200, 00128 Rome, Italy; m.bravi@policlinicocampus.it (M.B.); f.santacaterina@policlinicocampus.it (F.S.); 6 Physiotherapy Unit, CF Research Centre, Azienda Ospedaliera Universitaria Integrata di Verona (University Hospital of Verona), 37126 Verona, Italy; 7 Haute-École Arc Santé, HES-SO University of Applied Sciences and Arts Western Switzerland, 2800 Delémont, Switzerland; florian.forelli@he-arc.ch; 8 Orthopaedic Surgery Department, Clinic of Domont, Ramsay Health Care, OrthoLab, 95330 Domont, France; 9 Société Française des Masseurs-Kinésithérapeutes du Sport (SFMKS) Lab, 93380 Pierrefitte-sur-Seine, France

**Keywords:** anterior cruciate ligament reconstruction, hamstrings, open kinetic chain

## Abstract

**Background:** Hamstring tendon autografts are frequently used for anterior cruciate ligament reconstruction (ACLR), but they are associated with persistent hamstring strength deficits and delayed functional recovery. Current rehabilitation guidelines often delay open kinetic chain (OKC) hamstring exercises due to safety concerns, despite the limited supporting evidence. This uncontrolled, underpowered, and exploratory study aimed to evaluate the safety and effectiveness of introducing OKC hamstring strengthening exercises as early as three weeks after ACLR. **Methods:** An exploratory retrospective observational study was conducted at a single physiotherapy center on 13 patients (aged 18–35) who underwent primary ACLR with semitendinosus–gracilis grafts. Participants followed a standardized rehabilitation program including isometric leg curls at 60° and 90° knee flexion and long-lever glute bridges twice weekly, starting from postoperative week 3. Safety was assessed through predefined “safety flags” (pain > 4/10, hematoma, clinical hamstring strain). Strength outcomes, including isometric knee flexion strength at 60° and 90°, limb symmetry index (LSI), and endurance tests, were assessed at 6 and 12 weeks. **Results:** All participants completed the program without major adverse events. Pain remained consistently low (median 2.5/10), with only one transient episode exceeding the threshold. No other complications were recorded. Isometric knee flexion strength significantly improved between week 6 and week 12 at both 60° (*p* = 0.018) and 90° (*p* = 0.003), with large effect sizes. LSI at 90° also increased significantly (*p* = 0.006), whereas improvements at 60° did not reach significance. Endurance testing showed functional gains as early as 6 weeks. **Conclusions:** The early introduction of OKC hamstring strengthening exercises three weeks after ACLR with hamstring autografts appears safe and promotes clinically meaningful improvements in strength and endurance. These findings, while from a small uncontrolled study, challenge conservative rehabilitation protocols and support the reconsideration of early hamstring loading. Given the retrospective, uncontrolled, and underpowered design, these findings are hypothesis-generating and not generalizable beyond young adults with hamstring autografts; larger randomized trials are required.

## 1. Introduction

Anterior cruciate ligament (ACL) injuries are prevalent in athletes and are frequently associated with persistent functional limitations, sub-optimal return to previous performance levels, elevated reinjury risk, and, in some cases, shorter athletic careers [1,2,3]. In soccer, pooled data indicate that 72–90% of players resume competitive play; however, only 53–80% recover their pre-injury standard of performance [4,5,6]. The careers of soccer players after ACL injury last on average 3–8 years and are typically 1–2 years shorter than those of their uninjured peers [4,5,6]. Such persistent deficits after ACL injury may contribute to the growing reliance on surgical reconstruction (ACLR), whose incidence in the United States increased from 40.9 to 47.8 procedures per 10,000 individuals between 2004 and 2009 [7,8,9].

ACLR is mainly performed using either autografts, where tissue is harvested from the patient’s own body (commonly the hamstrings or patellar tendon), or allografts, where tissue is obtained from a donor. One of the key aspects after ACLR with hamstring tendon (HT) autograft is the harvesting procedure, which leads to persistent hamstring strength deficits commonly associated with loss of medial rotation, knee flexion, and varus strength [10]. The evidence for what kind of graft reduces the risk of re-rupture is not clear, but some authors suggest a slight increase in the risk for HT [11,12]. These findings underscore the necessity of incorporating adjunctive external stabilization techniques, such as lateral extra-articular tenodesis [13,14]. We can only hypothesize that the reported rise in graft re-rupture is linked to altered kinetic variables—such as a loss of knee-flexion range—but, although the current evidence does not support a definitive correlation, the impact of graft-harvesting techniques on the rotational stability and varus-moment control of the knee remains largely unexplored [15].

Hamstrings are crucial for dynamic knee stability and ACL protection [16,17]. In fact, hamstrings play an important role in knee joint stability by preventing medial femoral condyle lift-off, valgus opening, anterior tibial translation, and external rotation [16,18]. At mid- to long-term follow-up (1–2 years and beyond), a significant proportion of patients continue to experience measurable hamstring strength deficits compared to their uninjured limb or healthy controls [19,20,21,22]. Girdwood et al. [23] reported that knee flexor strength deficits of 5–7% compared to the contralateral limb are typical at 1 year, with little further improvement up to 5 years after ACLR. These deficits are even larger when compared to uninjured controls instead of the contralateral limb. Furthermore, a recent study reported limb symmetry index (LSI) values of 54%, 70%, and 76% for isometric knee flexor strength at 4, 8, and 12 weeks following ACLR using a hamstring graft [24]. These findings highlight the persistence of strength deficits in the early postoperative period after ACLR [24]. Moreover, hamstring weakness after ACLR tends to be more pronounced at deeper flexion angles (from >70° to max knee flexion) (i.e., shorter muscle lengths), during slower contraction speeds, and is especially evident when assessing rate of force development rather than peak strength alone [24]. After ACLR with HT, there is often a selective reduction in semitendinosus muscle thickness, with reported atrophy ranging from 10% to 28% [20]. After ACLR with hamstring graft, the semitendinosus often shows selective atrophy (10–28%), frequently accompanied by gracilis atrophy (~30%). If not compensated by hypertrophy of the semimembranosus or biceps femoris, this can lead to overall hamstring volume loss, reduced knee flexion strength, and imbalances between medial and lateral hamstrings that may impair transverse plane knee stability [21,25,26,27]. In addition to maximal strength, hamstring endurance is another clinically relevant aspect that may influence the postoperative recovery [28]. Endurance, defined as the ability to sustain repeated submaximal contractions over time, is crucial for functional knee stability during prolonged physical activity [29,30]. Importantly, endurance deficits may persist even when peak strength partially recovers, suggesting that monitoring endurance could provide complementary insights into muscle function after ACLR [31].

While rehabilitation protocols have evolved towards accelerated approaches [32,33] (which involve early unrestricted movement, immediate weight-bearing within limits, removal of immobilization braces, and a patient-centered goal of returning to high-level physical activity or sports within 6 months post-surgery), current guidelines often maintain a cautious approach to the early introduction of hamstring strengthening exercises, particularly open kinetic chain (OKC) leg curls, due to concerns about harvest site healing. Most research suggests waiting 6–8 weeks before starting to load the knee flexion [34,35,36].

However, this cautious approach is largely based on expert consensus [37] rather than high-level evidence [9]. At the same time, it is well established that early muscle activation is fundamental to counteract neural inhibition and muscle atrophy [38]. Given the persistence of hamstring strength deficits observed after ACLR with HT and the absence of robust data on the effects of early introduction of specific OKC exercises such as leg curls and bridges [20,37], there is a compelling need to reconsider the timing of their introduction. To date, no studies have specifically evaluated the safety and effectiveness of early OKC exercises on hamstring strength after ACLR. Seeking to address this gap in the literature, the present study aims to evaluate the potential for hamstring strength recovery associated with early OKC exercise implementation and analyze the incidence of potential adverse effects. We hypothesize that the early introduction of OKC exercises may facilitate faster recovery without increasing the risk of complications.

## 2. Materials and Methods

### 2.1. Study Design and Participants

An exploratory retrospective observational study was conducted in accordance with the Strengthening the Reporting of Observational Studies in Epidemiology (STROBE) guidelines [39].

This was a retrospective observational study conducted at a single outpatient physiotherapy center. The study followed the ethical principles outlined in the Declaration of Helsinki and its subsequent amendments. Ethical approval was obtained from the Institutional Review Board (Ethics Committee) of the Conseil d’Orientation Scientifique—GCS Ramsay Santé pour l’Enseignement et la Recherche (IORG0009085; IRB00010835; protocol code COS-RGDS-2025-06-002-FORELLI-F; approval date 11 June 2023), and all participants provided written informed consent for the use of their anonymized data.

We reviewed the records of 25 patients who underwent primary ACLR with autologous semitendinosus–gracilis grafts between December 2023 and December 2024 (all procedures were performed by the same surgical team). The inclusion criteria of an age range of 18–35 years and a Tegner Activity Scale score of ≥5 were chosen to focus on a population of physically active adults and athletes. This specific cohort is highly represented in ACL injuries and shares common rehabilitation goals, such as a high-level return to sport. Excluding pediatric and older adult patients helped to mitigate potential confounding factors related to age-dependent variations in muscle development, strength, and recovery rates.

The inclusion criteria were as follows:

- Age 18–35 years;
- Tegner Activity Scale ≥ 5;
- Isolated ACLR or combined with partial meniscectomy or lateral extra-articular tenodesis;
- Baseline knee range of motion ≥ 90° [40];
- Resting knee pain ≤ 3/10 on the Numeric Pain Rating Scale (NPRS);
- Absence of clinical signs of effusion or acute inflammatory flare (joint swelling < Trace on Stroke test compared to contralateral side, no redness or local heat or any other warning signs) [41].

The exclusion criteria were as follows

- Meniscal repair;
- Concomitant Posterior Cruciate Ligament or posterolateral corner injuries;
- Use of grafts other than hamstring tendons;
- ACL revision surgery;
- Additional ligament injuries requiring protection beyond standard ACLR rehab;
- Cartilage procedures requiring protected loading (e.g., microfracture);
- Clinically relevant postoperative complications before week 3 (e.g., infection, arthrofibrosis).

**Figure 1:**
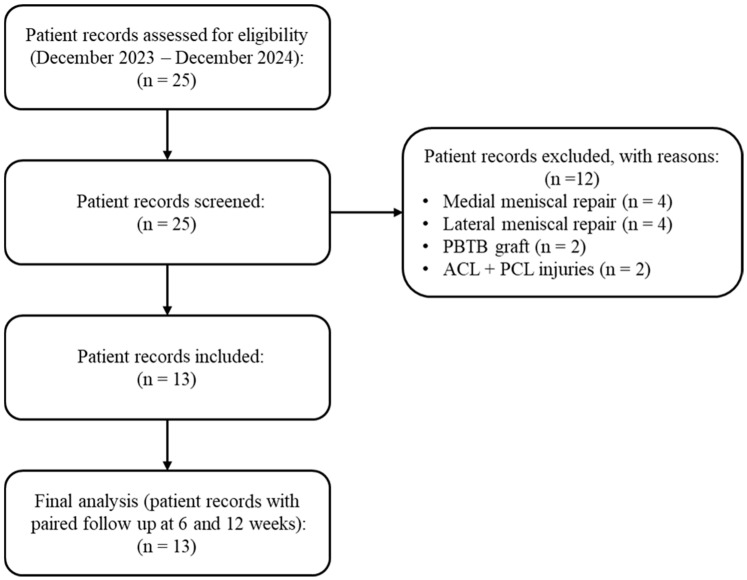
Flowchart of the retrospective selection process.

Of the 25 patient records reviewed, 12 were excluded due to concomitant procedures or alternative graft types, leaving 13 eligible records included in the final analysis (Figure 1). The same 13 participants were assessed at all timepoints, ensuring valid paired comparisons.

### 2.2. Rehabilitation Protocol

All participants began a hamstring strengthening protocol at 3 weeks after ACLR, two times per week. The protocol included 2 open kinetic chain exercises (leg curl) and 1 closed kinetic chain exercise (long lever glute bridge). During this period, no other isolated hamstring exercises were performed. Every patient followed a 3 times per week (every 48 h) strict rehabilitation protocol focusing on quadriceps, calf, core, hip, and balance and two hamstring exercises completed 2 times per week every 96 h (Table 1).

After 10′ of bike as warm-up, the intervention was structured in two primary phases: initially, OKC exercises were performed, followed by closed kinetic chain (CKC) exercises.

The first exercise was an isometric seated single leg curl on a leg curl machine at 90°, the second was an isometric seated leg curl on a leg curl machine at 60°, and the third was long lever glute bridges (patients lie supine with the knee flexed at 30°, foot in contact with the ground, and arms on the chest. For the seated leg curl machine, the range of motion (ROM) was standardized using the built-in lateral adjustment device, which allows the movement to be fixed at predefined joint angles (Seated Leg Curl R.O.M. machine, Technogym^®^, Cesena, Italy); for the glute bridge, we standardized the knee joint angle using a goniometer to ensure consistent ROM across participants. These exercises were completed at the beginning of each rehabilitation session.

Following the two hamstring primary exercises, participants completed a standardized protocol consisting of the following:

- Two quadriceps-focused exercises (leg extension and leg press),
- Two calf exercises (bent-knee and straight-knee calf raises),
- Two core stabilization exercises (plank and side plank),
- Two hip-focused exercises targeting the adductors and abductors (Copenhagen plank and lateral walk with a mini-band positioned at the ankles),
- One single leg balance exercise with eye closed.

From postoperative week 3 to week 6, every patient entered a three-week strengthening program consisting of three supervised sessions per week (nine sessions in total). All work was overseen and logged—sets × repetitions × external load or time-under-tension (TUT), plus any symptoms (including pain and/or burning sensation in harvest site or cramping in the posterior/medial side of the thigh), by the same physiotherapist, who has more than ten years’ experience in ACLR rehabilitation.

Each session began with quadriceps-dominant and multi-joint lower-limb exercises (e.g., leg extension, leg press). These drills followed a linear periodization scheme [42]: the earliest sessions used high repetitions with low external resistance, and subsequent sessions progressively reduced the repetition count while increasing the load, but only when the patient maintained full movement control throughout the range of motion.

Hamstring strengthening was scheduled twice per week. During those two sessions, patients performed three unilateral exercises: (i) an isometric leg curl hold at 90° of knee flexion, (ii) an isometric leg curl hold at 60° of knee flexion, and (iii) a long lever glute bridge. The initial prescription for each hamstring drill was five sets of forty-five-second holds, with thirty seconds of rest between sets; the injured limb was always trained first, followed by the uninjured side to avoid contralateral detraining. Starting loads were chosen to provoke neither pain with an NPRS less than 3 out 10 nor compensatory patterns like anterior pelvic tilt or feeling fatigue from the calf muscle. Quality was deemed acceptable when no anterior pelvic tilt (no lumbar lift-off from the backrest) appeared during the leg-curl holds and when the hip height remained stable during the glute bridge, with no height drop from the position reached at the beginning of the set. Whenever all sets met these criteria, with a numeric rating pain scale < 3, the next session’s external load was increased by ten per cent, and the TUT for each set was shortened by five seconds [43]. If a patient could not yet stabilize a single-leg glute bridge for the full TUT, the exercise began in the double-leg position and progressed to unilateral execution once a forty-five-second hold was achieved without loss of hip height.

The third weekly session omitted hamstring exercises but retained the same quadriceps and multi-joint progression, ensuring adequate recovery for the flexor muscles while maintaining overall lower-limb loading throughout the three-week block.

After 6 weeks, patients started a progressive isotonic slow resistance training with 0–90° range of motion in seated leg curl and full hip range of motion with knees at 30° of flexion on single leg long lever glutes bridge (Table 2).

For the isotonic seated leg curl and long lever glutes bridge, the strength progression was based on a linear periodization.

The initial load was 4 sets of 15 repetitions (with 2 repetitions in reverse) at a slow controlled tempo, and progression of the load was increased by 5–10%, depending on the pain tolerance (maximum NPRS score less than 3 out 10). With each load increase, repetitions were decreased by two (i.e., 15 → 12 → 10 → 8 → 6), while maintaining 4 sets and a 2 min rest between them. Once the patient reached 6 repetitions per set, the volume (sets and reps) was held constant, and only the external load was progressively increased.

### 2.3. Outcome Measures

The primary outcome is the safety and tolerability of the early introduction of OKC exercises. For each physiotherapy session, we searched the notes for three predefined “safety flags” recorded during the workout or within the following 24 h:

1. Pain rated > 4/10 on the NPRS.
2. Hematoma in the posterior thigh or at the graft-harvest site, described on inspection or palpation and, when necessary, confirmed by musculoskeletal ultrasound.
3. Clinical signs of hamstring-muscle strain, identified by acute localized pain in the posterior thigh or tendon, pain exacerbated by stretching or palpation, and, when suspected, confirmed by magnetic-resonance imaging [44].

Whenever one or more of these events appeared in the chart, the treating therapist halted the OCK component of the program. OKC work was restarted only after the patient was completely pain-free and never earlier than postoperative week 8, in line with our physiotherapy center guidelines.

The secondary outcome was the recovery of the hamstring muscle strength and endurance. Hamstring strength values recorded at postoperative week 3 served as the baseline, while measurements at weeks 6 and 12 were used to evaluate the progression of muscle strength over time. Before the test, patients underwent a standardized warm-up regimen, which consisted of 10 min on a stationary bike, followed by ten submaximal repetitions at 50% and 75%, and then one to two repetitions at 90% of their maximum voluntary contraction in the testing device [45,46].

At 6 weeks, the isometric strength of the hamstring muscles was tested at 90° and 60° of knee flexion using a fixed dynamometer with 1000 Hz frequency (K-push, Kinvent, France). The dynamometer force arm was calibrated according to the manufacturer’s guidelines before each testing session. The non-testing thigh was secured to the test bench with a strap at its mid-thigh level.

The force arm of the dynamometer was secured 2 cm proximal to the lateral malleolus to ensure a consistent lever arm across all participants. We calculated the lever arm for each participant by measuring the distance in meters between the lateral femoral condyle and the point where the dynamometer was placed on the lower leg, which was 2 cm proximal to the lateral malleolus. Patients were asked to push as hard and fast as they could for 5 s trying to avoid countermovement prior the start. In order to have a more detailed picture of the recovery, we also calculated the hamstring-to-quadriceps strength ratio. Quadriceps strength was assessed in a sitting position with the knee at 60° of flexion, in order to avoid stress on the graft [47]. The isometric test consisted of three 3 s maximal voluntary contractions with a 30 s rest interval [44,48]. The average of the three trials was calculated and used to compute the LSI.

We calculated the torque as the force, expressed in Newton normalized to the body weight, expressed in kilograms (N/kg) [49].

The endurance was tested through the glute bridge endurance test [31]: patients performed as many single-leg repetitions as possible in a supine position with the hip flexed at 45° and the knee at 90°, maintaining dorsiflexion [31]. Before starting the test, a target height was determined based on the patient’s maximal hip extension during a preliminary trial. The test was performed to the rhythm of 60 beats per minute. The test was stopped when the patient could no longer reach the target height or failed to maintain the rhythm. This test was chosen to assess both maximal and endurance strength of the posterior chain at the 6-week timepoint.

At the 12-week follow-up, all the same isometric strength tests were repeated, with the exception of the glute bridge endurance test, which was replaced by a 90°–90° isometric hamstring test (with both the hip and knee flexed at 90°) [44,48]. For both strength assessments test, the uninjured limb was tested first. Each isometric test consisted of three 3 s maximal voluntary contractions with a 30 s rest interval [44,48]. The average of the three trials was calculated and used to compute the LSI.

The LSI was computed through the following formula:

LSI = (Injured limb strength/Healthy limb strength) × 100.(1)

### 2.4. Statistical Analysis

All statistical analyses were conducted using JASP (Version 0.95.1; JASP Team, Amsterdam, The Netherlands). A descriptive analysis was performed for anthropometric and clinical characteristics. Normality tests were conducted for all variables with Shapiro–Wilk test and inspection of Q–Q plots. Given the small sample size (n = 13) and the limited power of normality testing, non-parametric methods were adopted regardless of the distributional results. When reported, safety outcomes (pain > 4/10, hematoma, clinical signs of strain) were summarized as counts and percentages. Continuous variables were reported as the median and interquartile range (IQR). A comparison analysis of differences in the hamstring isometric strength (60° and 90° knee flexion), quadriceps strength, hamstring-to-quadriceps ratio, and limb symmetry index (LSI) between week 6 and week 12 was performed using the Wilcoxon signed-rank test. The glute bridge endurance test was only descriptively reported (median [IQR]), as it was only performed at week 6. Effect sizes for paired non-parametric tests were expressed as the rank–biserial correlation (r), with 95% confidence intervals (CI). All statistical tests were two-tailed, with the significance level set at α = 0.05. No adjustment for multiple comparisons was applied, given the exploratory nature of the study. No a priori sample size calculation was performed; therefore, the analyses are underpowered, and the potential for type II error must be acknowledged when interpreting non-significant findings.

## 3. Results

### 3.1. Safety

All 13 participants (descriptive characteristics in Table 3) completed the rehabilitation protocol without interruption.

**Figure 2:**
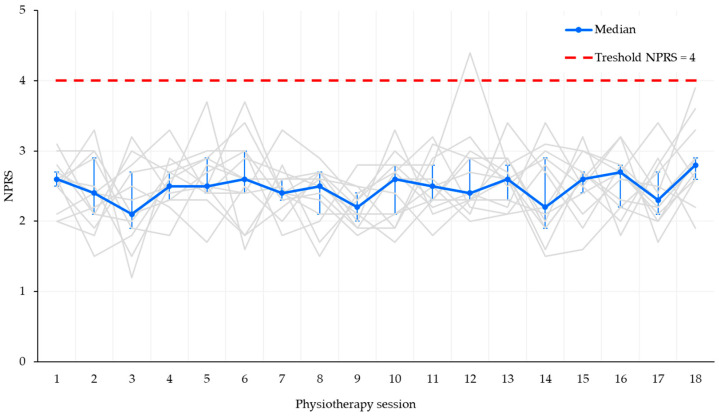
Individual pain trajectories (NPRS) recorded across the 18 supervised rehabilitation sessions. Thin grey lines represent single-patient values, while the blue line depicts the session-by-session median with interquartile range error bars. The dashed red line indicates the clinical threshold of 4 points.

Pain diaries documented consistently low discomfort levels throughout the 18 supervised sessions (234 observations in total). The median pain intensity recorded across all sessions was 2.5 (range 1.2–4.4). Inter-session values showed no clinically relevant differences, with most ratings remaining stably below the threshold of 4. Across the 234 monitored sessions, only one patient (7.7%) reported pain exceeding the clinical threshold (NPRS 4.4 at session 12). This was an isolated episode and did not require protocol interruption. No posterior thigh hematomas, graft site complications, or clinical signs of hamstring strain were observed. The distribution of pain scores across sessions and patients is illustrated in Figure 2.

### 3.2. Strength Recovery

**Figure 3:**
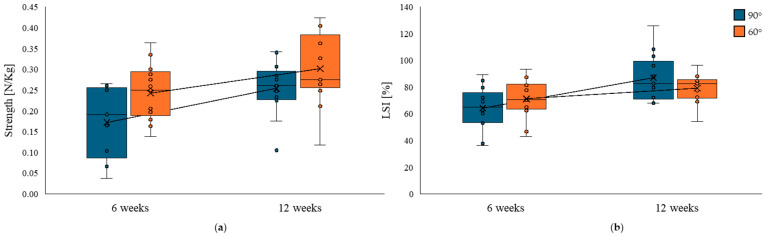
Distribution of quadriceps isometric strength measured at 90° (blue box plots) and 60° (orange box plots) of knee flexion in the ACLR limb at 6 and 12 weeks postoperatively. Box plots display the median, interquartile range, and individual data points; connecting lines represent individual trajectories between timepoints. An overall improvement in strength is observed in the operated limb, particularly at 90°, with inter-individual variability. The strength asymmetry between limbs appears to decrease over time. (**a**) show improvement in strength, (**b**) improvement in the limb symmetry index (LSI).

At 6 weeks, the isometric knee flexion strength of the operated limb was 0.25 N/kg (range 0.14–0.36) at 60° and 0.19 N/kg (range 0.04–0.27) at 90°. At 12 weeks, the strength increased to 0.28 N/kg (range 0.12–0.42) at 60° and 0.26 N/kg (range 0.10–0.34) at 90°. Paired comparisons between 6 and 12 weeks showed a significant improvement in isometric strength at both 60° (Z = −2.40, *p* = 0.018) and 90° (Z = −3.04, *p* = 0.003). The effect size, expressed as rank-biserial correlation suggested a large improvement both at 60° (r = −0.82, 95% CI −0.95 to −0.45) and 90° (r = −0.96, 95% CI −0.99 to −0.86). These results are illustrated in Figure 3.

The median LSI for isometric knee flexion tested at 60° was 70.4% (IQR: 42.9–93.4%) at 6 weeks, increasing to 82.4% (IQR: 54.2–96.3%) at 12 weeks. This difference was not statistically significant (*p* = 0.108). The effect size, expressed as the rank–biserial correlation, suggested a moderate improvement (r = −0.52, 95% CI: −0.83 to 0.05). The median LSI for isometric knee flexion tested at 90° was 65% (IQR: 36.4–29.2%) at 6 weeks, increasing to 82.7% (IQR: 67.9–126%) at 12 weeks. The Wilcoxon signed-rank test revealed a significant improvement in the LSI at 90° from 6 to 12 weeks (*p* = 0.006), with a large effect size (r = −0.82, 95% CI −0.95 to −0.50). Detailed descriptive and inferential statistics are reported in Table 4 and illustrated in Figure 3.

At 6 weeks, participants performed a median of 25 (IQR: 14–57) repetitions with the uninjured limb and 25 (IQR: 12–55) with the injured limb. The difference was statistically significant (Z = −2.43, *p* = 0.016), with a large effect size (r = −0.80, 95% CI −0.94 to −0.42). The corresponding LSI was 95.45%.

## 4. Discussion

The aims of this study were to evaluate the hamstring strength recovery and the safety of introducing both OKC and CKC hamstring exercises as early as three weeks after ACLR using a hamstring graft. The findings indicate that early implementation of these exercises appears to be safe and may promote improvements in hamstring strength and endurance, but these results should be interpreted with caution given the sample size and study design.

A previous study initiated hamstring-loading protocols at four weeks following ACLR with hamstring tendon autografts and documented progressive improvements in hamstring strength at 8 and 12 weeks, with an LSI of 69% and 75% at 60° of knee flexion, respectively; however, no significant gains were noted at 90° [47]. Greater deficits in knee flexion strength have been reported at deeper flexion angles (>70°), particularly in patients who underwent harvest of both semitendinosus and gracilis tendons, with residual weakness ranging from 3% to 27% even at 32 months after ACL [50]. Persistent weakness has also been observed at 18 months after ACLR, with LSIs as low as 60% at 90° and 70% at 70° of knee flexion in a prone position in the semitendinosus–gracilis group [51]. Moreover, one study documented reduced active knee flexion range of motion one year after surgery, suggesting ongoing deficits in muscular function and neuromuscular control [52]. Our results support the hypothesis that early introduction of both OKC and CKC hamstring exercises may counteract such long-term deficits [53]. No adverse events occurred during the intervention period. Patients reported only mild discomfort (median NPRS: 2.5) during or following training sessions, and no cases of hematoma, donor site complications, or hamstring strain were observed. These findings challenge the traditionally conservative approach of delaying OKC hamstring exercises after ACLR, a strategy primarily based on theoretical concerns rather than empirical evidence. Instead, our data suggest that earlier loading may be both safe and beneficial for optimizing the early recovery of hamstring strength and endurance.

### 4.1. Clinical Implications

The preliminary findings of this retrospective study suggest that early voluntary isometric and isotonic exercises, introduced at three weeks after ACLR in patients with isolated reconstructions and no meniscal repair, may be feasible and safe. While such early loading could potentially help counteract neural inhibition and support recovery of functional strength [54,55,56], these observations are based on a small sample and should be interpreted with caution. We cannot state whether natural healing, general exercise, or familiarizations could be plausible alternative explanations. Larger controlled studies are required before drawing firm conclusions about clinical implications [57].

### 4.2. Limitations

This study has several methodological and analytical limitations that must be acknowledged. First, given the small sample size, the study had limited statistical power. As such, non-significant findings cannot be interpreted as evidence of no effect, but rather as inconclusive results that require confirmation in larger cohorts. Second, the testing procedures were not fully consistent across time points, as different strength assessments were used, potentially affecting the comparability of the results over time. We decided to address endurance strength only at 6 weeks, because it is not considered a clinical milestone for 12 weeks assessment; so, we do not know whether the result achieved is consistent over time. Third, the chosen effect size metric may not have been the most appropriate for the type of data analyzed, which could lead to an over- or underestimation of the true magnitude of change. Fourth, some outcome measures were either insufficiently described or lacked clear operational definitions, reducing the transparency and reproducibility. In addition, safety outcomes were reported narratively rather than through systematic collection and quantification of adverse events, which limits the robustness of our safety claims. It is important to note that the small sample size and the narrative nature of the safety data collection limit the robustness of these findings. While the completion of 234 sessions with only one pain episode is a reassuring preliminary signal, this study is not powered to detect rare or uncommon adverse events; furthermore, because pain is subjective, and the source data were routine clinical records, outcome misclassification is possible. Rare adverse events cannot be excluded in a cohort of this size. Therefore, our safety claims should be interpreted as hypothesis-generating, with a need for larger prospective studies to systematically quantify and confirm the safety profile of this intervention. We also cannot address the effect on graft laxity due to the absence of the laxity test, but this was not an objective of the study. Another important limitation concerns the reliance on the LSI as the primary marker of recovery: while useful, LSI does not capture absolute strength values and may therefore introduce bias, especially if the contralateral limb is also affected by deconditioning. Moreover, the retrospective design without a control group, the small sample size (n = 13), single surgical team, single rehabilitation center, and standardized treatment by the same therapist limit the internal validity. Selection and reporting biases are possible, and causal inference is not warranted. The external validity is restricted to young physically active adults (18–35 years) who underwent primary ACLR with hamstring autografts without meniscal repair. Finally, the absence of a control group prevents any causal inferences regarding the superiority of early hamstring loading over standard rehabilitation protocols and limits the generalizability of the findings. Collectively, these limitations highlight the preliminary nature of our results and underscore the need for future prospective randomized controlled trials with larger sample sizes, standardized outcome measures, systematic safety reporting, and long-term follow-up to confirm and expand upon these observations.

### 4.3. Future Directions

Future studies should build on the present findings by prospectively testing whether the early introduction of voluntary isometric and isotonic hamstring exercises at three weeks post-ACLR leads to superior recovery of hamstring strength compared with standard protocols. Randomized controlled trials with adequately powered sample sizes are warranted to evaluate not only the LSI but also the absolute strength values, functional performance measures, and long-term return-to-sport and re-injury outcomes. Methodological refinements should include the use of consistent strength assessments across time points, systematic collection of adverse events to better characterize safety, and appropriate effect size metrics to capture the magnitude of change. Additionally, future research should explore whether the timing and intensity of early strengthening differentially affect patients with various graft types or concomitant procedures and whether targeted interventions can mitigate the atherogenic muscle inhibition observed in this population.

## 5. Conclusions

Introducing open-kinetic-chain hamstring strengthening from week 3 post-ACLR with a hamstring autograft appeared feasible and was associated with short-term improvements in inter-limb strength symmetry and endurance. No adverse events were observed, and meaningful improvements in inter-limb strength symmetry and endurance were obtained. These findings challenge the traditionally conservative approach and provide a rationale for reconsidering early hamstring loading in postoperative rehabilitation protocols. These preliminary observations should be interpreted with caution and require confirmation in adequately powered randomized controlled trials with standardized safety monitoring and longer follow-up.

## Figures and Tables

**Table 1:** Structure of the rehabilitation program weeks 3–6.

Day 1	Day 2	Day 3
10′ bike	10′ bike	10′ bike
Seated hamstring isometric curl at 90°	Horizontal single leg press	Seated hamstring isometric curl at 90°
Seated hamstring isometric curl at 60°		Seated hamstring isometric curl at 60°
Isometric long lever glute bridge	Seated leg isometric leg extensions	Isometric long lever glute bridge
Horizontal single leg press	Standing calf raise	Horizontal single leg press
Seated leg isometric leg extensions	Bent knee calf raise	Seated leg isometric leg extensions
Standing calf raise	Lateral walk with mini band	Standing calf raise
Bent knee calf raise	Adductor plank	Bent knee calf raise
Lateral walk with mini band	Single leg balance exercise with eyes closed	Lateral walk with mini band
Adductor plank		Adductor plank
Single leg balance exercise with eyes closed		Single leg balance exercise with eyes closed

**Table 2:** Structure of the rehabilitation program weeks 6–12.

Day 1	Day 2	Day 3
10′ bike	10′ bike	10′ bike
Seated isotonic leg curl	Horizontal single leg press	Seated isotonic leg curl
Isotonic long lever glute bridge	Seated leg isometric leg extensions	Isotonic long lever glute bridge
Horizontal single leg press	Standing calf raise	Horizontal single leg press
Seated leg isometric leg extensions	Bent knee calf raise	Seated leg isometric leg extensions
Standing calf raise	Lateral walk with mini band	Standing calf raise
Bent knee calf raise	Adductor plank	Bent knee calf raise
Lateral walk with mini band	Single leg balance exercise with eyes closed	Lateral walk with mini band
Adductor plank		Adductor plank
Single leg balance exercise with eyes closed		Single leg balance exercise with eyes closed

**Table 3:** Demographic data.

Characteristics	ACL Patients (n = 13)
Age ^1^	27 (25–30)
*Gender*	
Male, n (%)	9 (69.2)
Female, n (%)	4 (30.8)
Weight (Kg) ^1^	71 (68–79)
Height (cm) ^1^	178 (172–183)
*Graft type*	
R HT/G, n (%)	6
L HT/G, n (%)	7
Time from surgery (weeks)	21 (19–22)

^1^ Data are reported as median (IQR). Yr = years; HT = hamstring tendons, Kg = kilograms.

**Table 4:** Limb symmetry index at 6 and 12 weeks post-ACLR. Hamstring strength tests performed in a seated position at 60° and 90° of knee flexion.

Isometric Hamstring Muscle Test	LSI at 6 Weeks Median [IQR]	LSI at 12 Weeks Median [IQR]	Δ LSI	*Z*	*p*-Value	r	95% Confidence Interval
Knee flexion = 60°	70.4% [42.9–93.4]	82.4% [54.2–96.3]	+9.5%	−1.64	0.108	−0.52	−0.83 to 0.05
Knee flexion = 90°	65% [36.4–89.2]	82.7% [67.9–126]	+19.5%	−2.62	0.006	−0.82	−0.95 to −0.50

Note: IQR = interquartile range; Δ LSI = change in limb symmetry index between 6 and 12 weeks. Wilcoxon signed-rank test was used. r = effect size calculated using rank–biserial correlation.

## Data Availability

The data supporting the findings of this study are available from the authors upon request.
